# Neoliberalism in academia: reflections from a parasitologist

**DOI:** 10.1186/s13071-024-06574-1

**Published:** 2024-11-24

**Authors:** Robin B. Gasser

**Affiliations:** Melbourne Veterinary School, The University of Melbourne, Parkville, VIC 3010 Australia

**Keywords:** Neoliberalism, Managerialism, Impact, Academia, Higher education, Universities

## Abstract

This article examines the major transformation within the higher education sector, specifically the shift from traditional academia to neoliberal academia, with an emphasis on its impact on academics who entered the field in the 2000s. Many of these individuals may not fully recognise the extensive political and structural changes driven by neoliberalism over the past 2 decades. Published literature shows how the widespread adoption of managerialism in a neoliberal context—particularly in the Anglo-Saxon world—has markedly altered the academic landscape. This shift has led to the marketisation of education, characterised by increased student tuition fees, performance metrics and a change in academic values, including professional autonomy and academic freedom. The present article further explores how these alterations have affected the wellbeing of academics, particularly early- and mid-career scholars, by institutions prioritising economic efficiency over intellectual enquiry, increasing administrative workloads and promoting a consumerist model of education. Drawing on both evidence from the peer-reviewed literature and experiences, the implications of these changes for academic careers, job satisfaction and the broader mission of universities as centres of scholarship and public service are discussed. The article concludes with a call to action for academic leaders and policymakers to recognise and address challenges posed by neoliberalism and managerialism, emphasising the need to support and protect the core values of academia in the face of ongoing changes.

**Graphical Abstract:**

**Figure:**
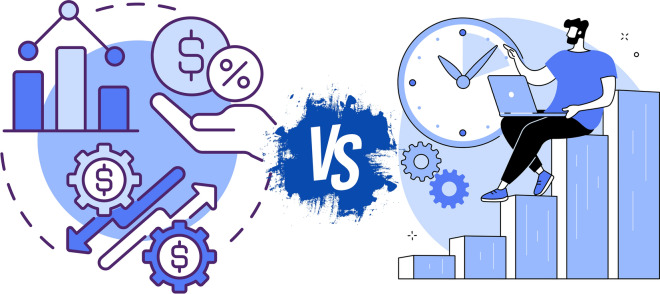

## Background

Being an academic is a privilege and comes with many rewarding experiences and opportunities. Some of the great things are meant to include intellectual freedom; working in areas of passion; life-long learning; contributing to society through education, research and engagement; collaborating with experts from around the globe; and mentoring students and staff, guiding their development and inspiring future leaders. Like many of my contemporaries, I have been fortunate to have enjoyed these great things in my role as a university academic working in the field of Parasitology. However, since the late 1980s, we have observed and experienced some significant changes in the higher education sector, both nationally and internationally, that are challenging the meaning, role and values of traditional academia [1] and potentially limiting academic performance, practice and genuine career development [2–5].

It has taken me “a while” to realise the impact that these changes are having or have had; fortunately, while in lockdown during the COVID-19 pandemic, I had the opportunity to ponder and read about (what colleagues in various disciplines were writing and saying) regarding changes to higher education since the 1990s. I reviewed and appraised some of the facts, figures and analyses about why and how these changes have come about. I should have done this much earlier, but, as is usual for some of us academics, we tend to concentrate on work challenges at hand and commit to contributing to our chosen disciplines and often do not have the luxury of time to think and philosophise deeply about topic areas that relate more broadly to our roles, duties and functions and to make real sense of signs or signals that we notice are “not quite right”. I feel that now is a good time to share some of my findings particularly with younger colleagues (i.e. early-career academics) in my discipline area, both nationally and internationally, because many of them appear to be unaware of the changes that have occurred in the evolution of the higher education sector over the past decades. My intent here is to share some information and thoughts to create awareness. This is an analysis, not a critique.

Expanding on a recent Science & Society brief [5], in the present article, I provide some background on liberalism and neoliberalism, and the transformation of the higher education system in the late 1980s, with a particular focus on the higher education sector in Australia, reflecting on similar situations in other countries. I explain how changes brought about by neoliberalism and associated managerialism can affect academics’ careers, progress, career success and life, as well as the institutions that they work in and care about. The purpose of this piece is to inform particularly early and mid-career academics in our discipline of Parasitology and in other Science, Technology, Engineering, Mathematics and Medicine (STEMM) and Humanities, Arts and Social Sciences (HASS) areas. It focuses on the Australian context, but with parallels in some other, particularly English-speaking, countries such as the UK and the USA.

### Liberalism

Liberalism is a philosophical and political ideology that has evolved over several centuries, and its development can be traced through several key historical phases including (1) the Enlightenment (seventeenth-eighteenth century), (2) Classical Liberalism (18th-19th century), (3) Liberal Reforms and Social Liberalism (19th–20th century) and (4) Modern Liberalism (20th century–present) [6].

Liberalism emphasises individual rights, equality, democracy and the protection of civil liberties; it advocates for a political system in which individuals have the freedom to pursue their own lives, make choices and express their views, with minimal interference from the state, while ensuring that everyone has equal rights under the law. Liberalism has evolved over time, and there are different strands within the ideology, including: (i) classical liberalism, which focuses on limiting government power and maximising individual freedom, and (ii) social liberalism, which supports a more active role for the state in addressing social inequalities and ensuring a fair distribution of resources.

In the context of liberalism, universities are characterised by key principles and practices that reflect the ideology’s emphasis on individual freedom, equality and the pursuit of knowledge [1]. Some of the key characteristics of universities in a liberal framework include academic freedom; autonomy; broad and inclusive education; equal access and opportunity for all individuals; promotion of critical thinking and debate; civic engagement and social responsibility; public service and knowledge dissemination; diversity and inclusion; and secularism [7]. These characteristics collectively reflect the commitment to fostering free inquiry, advancing knowledge and contributing to the development of informed, engaged and socially responsible individuals. These values of liberalism were instrumental in shaping the academic freedom and critical thinking that were characteristic of universities in the 20th Century.

Throughout its history, liberalism has been a dynamic and evolving ideology, adapting to the challenges of each era, while maintaining a core commitment to individual freedom, equality and democratic governance. The twentieth century saw an expansion of liberal principles, including the promotion of civil rights, gender equality and human rights [8]. In the late twentieth century, neoliberalism emerged as a variant of liberalism, advocating for free-market capitalism, deregulation and a reduction in government spending [8].

### Neoliberalism

Neoliberalism is a political and/or an economic ideology that emphasises the efficiency of free markets, the significance of individual entrepreneurial freedom and the reduction of government intervention in the economy [8]. It advocates for policies such as deregulation, the privatisation of state-owned enterprises, the reduction of public spending, free trade and the promotion of economic growth that is led by the private sector and market forces. In the context of neoliberalism, universities are characterised by key features that reflect the broader neoliberal ideology, which emphasises market efficiency, individual responsibility and a reduced role for the state in economic and social life. Key characteristics of higher education in a neoliberal context include:

- (i) Privatisation and reduced public funding. The reduction in public funding for higher education shifts towards private funding sources, including tuition fees, corporate partnerships and philanthropy. Neoliberal policies encourage the privatisation of some university services and the involvement of the private sector in funding and supporting research, leading to partnerships with corporations.
- (ii) Marketisation of education as a commodity, with students being viewed as consumers purchasing a service [9]. This has led to a focus on branding, marketing and student satisfaction metrics as key performance indicators, and a shift towards higher tuition fees, with students bearing a significant share of the costs associated with their education—justified by viewing education as a personal investment that yields future economic returns.
- (iii) Performance metrics and accountability. Universities are increasingly held accountable through performance metrics such as graduation rates, employment outcomes, student satisfaction surveys and research productivity. These metrics are used to evaluate institutional success and guide funding decisions. Neoliberalism also encourages competition among universities, often driven by global rankings that prioritise metrics including research output, employability of graduates and internationalisation (e.g. [10]).
- (iv) Commercialisation of research. Research agendas are increasingly driven by translation, commercialisation and practical application, often aligned with industry needs. This leads to a prioritisation of research that has economic benefits over fundamental and theoretical discovery. Universities might emphasise the generation and capture of intellectual property and patents as sources of revenue, often partnering with private companies to develop and commercialise innovations.
- (v) Managerialism and corporate governance. In a neoliberal context, universities adopt corporate management practices, with a focus on efficiency, cost-cutting and maximising returns on investments. This includes the adoption of strategic planning, performance management and managerial oversight. Governance often shifts to a more centralised, top-down approach, with decisions made by administrators rather than academics, which can reduce the influence of faculty members and alters the traditional collegial governance model [2, 3, 11].
- (vi) Casualisation of the academic labour force. Neoliberalism has contributed significantly to the casualisation of the academic workforce, with an increasing reliance on adjuncts, part-time faculty and contract staff. This had led to reduced job security and benefits for many academics, leading to a more stratified and precarious workforce [12, 13]. Academics can face greater pressure to perform in teaching, research and/or service roles, often with reduced support, which can lead to increased workloads and stress. In addition, in some universities, senior academics are increasingly being replaced by junior staff who, often burdened with heavy teaching responsibilities, may struggle to thrive without a more balanced portfolio.
- (vii) Shift in educational priorities. Universities may prioritise programs and courses that align with market needs and have higher employability outcomes. Neoliberal policies can lead to the standardisation of curricula and teaching practices, aiming for efficiency and scalability, which can reduce creativity and responsiveness to diverse student needs [14].
- (viii) Students as marketplace consumers. The relationship between students and universities is increasingly framed as a consumer relationship, where students are seen as customers whose satisfaction is paramount. This can lead to a focus on providing amenities and services that enhance the student experience, sometimes at the expense of academic rigour. To attract and retain fee-paying students, some universities have been accused of “loosening” entry requirements and inflating examination scores; some critics have argued that this approach has led to the devaluation of degrees, potentially lowering the competence of graduates, damaging the reputation of higher education and having societal impacts (e.g. [2, 9]). Students often graduate with significant debt due to higher tuition fees and reduced public support, which means that many students view their education in terms of return on investment [9].
- (ix) Globalisation and international competition. Neoliberal policies encourage universities to recruit international students as a source of revenue from tuition fees, which often leads to competition for students from abroad, with universities tailoring programs and services to attract them [15]. In this context, universities may form international partnerships and networks to enhance their global reputation and expand their market reach.
- (x) From public to private good. Neoliberalism shifts the focus of higher education from serving the public good to providing private benefits to individuals, potentially diminishing the role of universities as institutions that contribute to societal well-being, democratic citizenship and social equity.

These ten characteristics reflect a broader influence of neoliberal ideology on higher education, leading to significant changes in how universities operate, how education is valued and how academics and students engage with, and relate to, their institutions. Critics argue that these changes undermine the traditional mission of universities as centres of critical enquiry and public service, prioritising economic efficiency and market-driven goals over intellectual and social ideals [2, 3, 13, 16–20].

### Higher education transformation at the end of the twentieth century

Lake et al. [21] provided an informative account of the humanistic tradition of education from Classical Antiquity through to the present, as the cultivation of the whole person as an individual and as a responsible citizen, which arguably still underpins European and some other western education systems, but which, according to the authors, “has been eroded”. These authors also provided a history of universities in Australia from 1850 to the present, emphasising challenges that now face the higher education sector, some of which have been exacerbated, but not caused by the COVID-19 pandemic. The pandemic has been a stark reminder of the critical importance of areas such as epidemiology and public health, which were not prioritised under neoliberal funding regimes.

Of relevance to the present paper is the transition from liberalism to neoliberalism. Like developments in some other Anglo-Saxon countries, such as the USA during Reaganomics and the UK under Thatcherism [22], Australia's higher education sector has undergone significant changes. There was a profound transformation under the politician John Dawkins, who served as Minister for Education from 1987 to 1991 under a Labor government. This period was marked by several driving concerns, including the pressures of globalisation, the growing integration of technology in the workplace, international shifts from manufacturing to a post-industrial “knowledge society” and Australia's lagging international competitiveness [23, 24]. Additionally, there was a renewed focus on supporting economic growth, responding to the 1982–83 recession, addressing tensions within the binary system of Universities and the Colleges of Advanced Education (CAEs) and adapting to broader trends toward the privatisation of public services [21]. These concerns were compounded by public sector reforms that involved significant cuts to public funding and government restructuring, all of which inevitably impacted universities.

Dawkins’ approach to reform was characterised by a “top-down approach”, with limited genuine consultation, largely guided by the findings of the 1985 Review of Efficiency and Effectiveness in Higher Education. In 1987, he introduced a “Green discussion paper”, which was followed 6 months later, in 1988, by an amended “White policy paper”. Central to Dawkins’ vision [25] was the restructuring of the university sector to create fewer but larger institutions. The federal education department was to negotiate a specific profile of activities with each university, aligning them with national priorities and allocating funding accordingly. The competition for public research funding would be centralised through the Australian Research Council (ARC), established in 1988. Concurrently, the government began to relinquish tight control over the internal governance of universities—a process that increasingly transferred power to Vice-Chancellors, Boards and the newly expanded managerial roles of Pro-Vice Chancellors (PVCs), Deputy-Vice Chancellors (DVCs) and Deans, through state-based enabling legislation.

The implementation of these reforms was swift, with the number of institutions decreasing from 18 universities and 47 CAEs in 1985 to 30 universities by 1991, to 35 by 1995 and to 42 by 2024 (ref. [21]; https://en.wikipedia.org/wiki/List_of_universities_in_Australia). Although the changes sparked considerable debate over the years (e.g. [26–34]), criticisms largely failed to bring about significant alterations to the reform process. While some issues became apparent early on, the broader consequences of the Dawkins' reforms became conspicuous during and after the COVID-19 pandemic. Interestingly, subsequent governmental and external reviews of these reforms, such as those by Croucher et al. [35] (2013), did not seem to recognise many of the deeper implications of the Dawkins “reform” as problematic (cf. [21]).

A key goal of the policies of Dawkins [36] was to mandate a review of how institutions were managed, aiming to establish robust systems for accountability and performance evaluations. The Dawkins White Paper [37] anticipated modifications in accounting procedures, the introduction of performance metrics and the refinement of audit practices linked to higher education funding and university profiling (i.e. the process of evaluating and categorising universities based on various performance indicators, including research output, teaching quality, financial stability and student satisfaction). Furthermore, research funding was increasingly directed through competitive grant programs (e.g. ARC and the National Health and Medical Research Council of Australia [NHMRC]), designed to "maximise the research potential of the Australian Higher Education System and align more closely with broader national goals" [38]. As a result, the role of research within this system was redefined in the 1990s, with funding being allocated based on principles of competition.

The next significant reform in higher education followed the Hoare Review [39], which advocated for universities to adopt new governance structures, managerial capabilities and workplace practices. Implementing these recommendations resulted in cost reductions, enhanced resource management—including the imperative for universities to diversify their income streams beyond government funding—and establishment of accountability and performance assessment mechanisms. These assessments were based on university objectives and performance indicators across various operational areas [40, 41]. Centralised management practices were also introduced, including the appointment of senior managers with expertise in finance, human resources and strategic planning. This shift toward a "professionalised" managerial system, borrowed from the private sector, curtailed academic authority and collegial decision-making processes [18, 31, 42]. Consequently, managerialism began to take hold, leading to power struggles and a division between academics and administrators, particularly concerning finance and resource allocation, but also regarding academic functions and activities (cf. [11, 42, 43]).

In the 1990s, universities increasingly adopted business accounting and management control techniques, with performance evaluations focusing more on economic and financial outcomes than on social impacts [41]. The pressure to diversify income sources beyond government funding spurred the creation of quasi-markets within higher education. This, in turn, heightened competition among staff and has since placed considerable pressure on academic staff to secure research grants and fellowships through competitive and other external funding schemes (e.g. [13]).

From 2001 to 2024, the landscape of research funding in Australia’s higher education sector underwent a profound transformation, marked by shifts in policies, priorities and mechanisms for resource distribution. This evolution built on the foundation laid in 1988 with the establishment of the ARC in response to the Dawkins White Paper and later was solidified when the ARC became an independent body in 2001 under the Australian Research Council Act.

One of the most notable changes during this period was the shift toward competitive funding for research, where resources were increasingly tied to performance indicators, including publication output, citation impact and successful grant applications. The National Competitive Grants Program (NCGP) became central to this approach, funding research projects that demonstrated both merit and alignment with national priorities. Simultaneously, there was a growing emphasis on aligning research funding with government priorities. This meant that projects focusing on areas of strategic national importance—such as health, technology and sustainability—were more likely to receive funding than other areas. The ARC’s Strategic Research Priorities and National Research Priorities played a pivotal role in guiding this allocation process, ensuring that research efforts were aligned with the government's objectives. This period also saw an increasing focus on impact and accountability. It was no longer sufficient for research to be published in academic journals; there was a strong push to demonstrate how public investment in research translated into tangible societal benefits. This led to the introduction of initiatives such as the Excellence in Research for Australia (ERA) assessment and the Engagement and Impact Assessment (EIA), which aimed to evaluate not just the quality of research, but also its broader impact on society. These assessments influenced future funding decisions, further embedding “research impact” in the funding landscape. Additionally, universities and research institutions were encouraged to diversify their funding sources. Beyond relying on government grants, there was a push to seek funding from industry, philanthropic partners and diverse international sources. Programs such as the ARC-embedded Linkage Infrastructure, Equipment and Facilities (LIEF) scheme facilitated this diversification by offering co-funding opportunities for research infrastructure, allowing institutions to build stronger partnerships and secure more varied financial support. This reconfiguration of research funding not only reshaped how resources were distributed but also influenced the very nature of research conducted in Australian universities, driving them to align more closely with national priorities and societal needs, while fostering accountability and impact.

An illustrative example of the “need for alignment” was an incident that made headlines in Australia in 2021, when it emerged that, under a Liberal-National Coalition government, the then Federal Minister for Education, Stuart Robert, vetoed six ARC grants (https://www.afr.com/policy/health-and-education/more-than-60-professors-protest-stuart-robert-s-research-grant-veto-20220110-p59n5t); these grants, which had already been peer-reviewed and approved by the ARC, were later disallowed by the minister for not aligning with perceived "national priorities”. Fortunately, under the ensuing Labor government, the Australian Research Council Act was amended in 2024 (https://parlinfo.aph.gov.au/parlInfo/search/display/display.w3p;query=Id:%22legislation/bills/r7130_aspassed/0000%22), such that Ministers would no longer be permitted to veto approved grants, ending “political interference”.

### Managerialism in a neoliberal context

Although there are various definitions, managerialism often refers to the application of managerial techniques, values and ideologies across sectors, particularly within public institutions such as universities. In a neoliberal society, managerialism is deeply intertwined with market-oriented reforms, where the “logic” of the market—focused on efficiency, competition and profitability—is imposed on organisations that were traditionally governed by distinct principles, such as the humanistic pursuit of knowledge, professional autonomy and public service (cf. [8, 11, 43]).

Key characteristics of managerialism include:

(i) Market-oriented reforms: Under neoliberalism, public institutions are restructured and run like market operations, emphasising competition and the pursuit of economic efficiency. Managerialism supports this focus by introducing practices such as performance-based funding, where success is measured by financial performance and output metrics.
(ii) Performance measurement: Managerialism is characterized by a focus on metrics, benchmarks and key performance indicators (KPIs). These metrics often prioritise quantifiable outputs, such as numbers of publication or graduation rates over the genuine quality of research or education. This leads to a reductionist approach to evaluating success, where what can be readily measured is valued more than what is not.
(iii) Centralised control: Managerialism centralises decision-making to professional managers, often at the expense of the autonomy of academics. This shift can undermine traditional governance structures that used to exist in universities under liberalism, where decisions were expertise-driven or collegial. A proliferation of centralised administrative positions, driven partly by a perceived need for increased oversight and audits, has coincided with reductions in technical and local support staff who could alleviate work burdens on senior academics. Such roles are often undervalued, as they are not seen as revenue-generating.
(iv) Cost-cutting and efficiency: Managerialism aligns with neoliberalism’s emphasis on austerity, often leading to cost-cutting measures, including downsizing, outsourcing and restructuring. These measures are “justified” by the perceived need to reduce public spending and to increase efficiency, often without regard for adverse impact(s) on the quality of services, research, education and/or the well-being of employees.
(v) Accountability and auditing: The rise of managerialism is associated with an increased focus on accountability, operationalised through continuous auditing, performance evaluations and reporting. While accountability is important, its implementation under managerialism can lead to a culture of surveillance and control, where the trust in academic judgement is eroded.

Under the influence of neoliberalism, managerialism has become entrenched within universities in Australia and other countries predominantly in the Anglo-Saxon world. In Australia, this shift has enabled the Federal Government to exercise "governance at a distance" by leveraging neoliberal strategies that promote centralised management within these institutions [44]. The top-down approach typical of managerialism, coupled with performance measurement practices, permeates all levels of a university’s operation—from the executive management down to individual academics [42, 44]. Furthermore, neoliberal governance has created indirect methods and approaches to facilitate the control of individuals without assuming direct responsibility for them [45]. These practices have positioned academics as self-managing entities—apparently "free agents" who, while appearing autonomous, are guided by overarching policy norms and regulatory frameworks [46].

### Enhanced emphasis on metric-based assessments

The ascendance of managerialism and the emphasis on performance evaluations can be attributed to an erosion of trust between public universities and the government [42, 47]. In Australia, this shift has been linked to the implementation by government of stringent performance criteria and accountability frameworks, particularly via research assessment exercises (RAEs), such as the Excellence in Research for Australia (ERA), introduced by the Federal Labor Government in 2008 [42]. RAEs have become part of the academic landscape in some countries, such as Australia, where they serve to evaluate the quality (and quantity) of research produced by universities, with funding allocated based on these assessments. Although the intent behind RAEs is to “drive” excellence and ensure accountability in the use of public funds, their impact on universities and academic staff has been the subject of considerable debate.

For universities, RAEs significantly influence the allocation of government funding, prompting institutions to compete and to strive to improve their research outputs and rankings in league tables. This competitive environment can lead universities to prioritise research areas that are likely to score highly in these assessments, sometimes at the expense of a broader academic focus. As a result, disciplines such as the humanities and social sciences (HASS), which are less likely to achieve high ratings, usually become underfunded and marginalised. Moreover, the focus on research quality has driven universities to adopt increasingly strategic approaches to managing research (i.e. performance management systems) at the university, faculty, departmental, group and/or individual levels [48, 49]. Institutions have developed policies aimed at enhancing research productivity, such as incentivising publications in prestigious journals, attracting leading researchers and retaining them, provided they keep complying with performance criteria. This shift has also influenced university governance, with management structures often prioritising research performance over other crucial academic activities, such as teaching and community engagement. The outcomes of RAEs also contribute to the global reputation of universities, effecting their ability to attract international students and staff. High performance in these assessments can significantly elevate a university’s standing in global rankings, an aspect that prospective students and staff frequently consider when choosing institutions in which to study.

For academic staff, the pressure induced by RAEs can manifest in several ways. The demand to publish in high-impact journals and secure research funding from diverse sources has markedly increased workloads, creating an environment where the quantity of research outputs might sometimes be prioritised over quality. This shift has contributed to a culture of stress and burnout among many academics [50–52] and has an unfortunate but foreseeable consequence—a rise in research integrity concerns [53], which threatens the very integrity of science.

Furthermore, RAEs have heightened the importance of research performance for career progression, with staff who fail to meet expected research outputs facing challenges in securing promotions and/or retaining their positions. This is particularly challenging for early and mid-career researchers, who may struggle to establish themselves in a competitive environment where established academics with strong track records and research teams are more likely to be supported by government bodies (e.g. ARC and NHMRC), particularly in an environment where the overall success rates for national-competitive grants and fellowships is increasingly < 10%.

Additionally, the focus on research metrics can lead to a sense of alienation among some academic staff, who may feel that their broader contributions to academia, such as teaching and mentorship, are undervalued [54]. The pressure to conform to institutional or government research priorities can also diminish academic autonomy, discouraging staff from pursuing their own scholarly interests [48], and compromises important but niche disciplines and research areas that are “not on the list”. While the implementation of ERA exercises in Australia (in 2010, 2012, 2015 and 2018) was intended to lead to improvements in research quality and accountability, it has also introduced challenges that need to be addressed to attain and maintain a balanced and sustainable academic environment in which academic freedom and staff well-being, research excellence and career development are priorities.

### Financial vulnerability of universities

Under the influence of neoliberalism and managerialism, many universities have increasingly had to rely on tuition fees from students and external funding sources as key elements of their financial strategies, often supplementing or even replacing government support. Although many institutions have sustained their operations and competed on a global scale, this context has financial vulnerabilities, particularly when external factors disrupt revenue streams.

Recently, Guthrie et al. [55] studied financial data of Australian universities from 2009 to 2019. Their analysis revealed that, while universities are viable, in terms of investments and revenue, they face the risk of financial vulnerability if they do not receive full government funding for research; currently, universities subsidise their research expenditures using revenues from teaching. In another study, Carnegie et al. [56] identified the strategies of Australian universities to recruit large numbers of students from select countries (“big markets”), such as China, as high-risk, and highlighted low admission standards for international students at some universities, including members of the prestigious “Group of 8”. These authors indicated that sensitive political relationships with some countries, coupled with possible revenue declines linked to stringent admission quality, exposes Australian universities to significant financial and social risks because of their heavy reliance on such overseas markets.

The COVID-19 pandemic highlighted the risks associated with the current high dependence on tuition fees paid by international students [42, 57]. As global travel restrictions and health concerns reduced student mobility, many universities experienced a significant decline in international enrolments, leading to major financial shortfalls in most cases. The response of universities was to substantially and rapidly cut costs and jobs (estimate: *n* ≥ 40,000 in Australia), with other major ramifications for society, including altered employment conditions, mental health issues in students, academics and other staff, limitations of online teaching/learning, and a lack of a genuine (i.e. traditional) university experience for students. This situation underscores the need for governments and universities to reassess the reliance on tuition fees from fee-paying students.

Another recent example is the sudden change in the student visa policy by Federal Government of Australia, with a cap at 270,000 individuals entering Australia (https://www.insidehighered.com/news/quick-takes/2024/08/28/australia-caps-international-student-enrollment). Stricter visa conditions and modifications to post-study work rights will likely make Australia a less attractive destination for international students, thereby reducing overseas student numbers and threatening a key source of revenue for most universities in this country. These are some examples and developments that emphasise the financial vulnerability of the higher education sector in this country and the importance of a solid financial base that can mitigate the impact of external variables, such as a pandemic or an epidemic, policy changes and other potentially complex socio-political factors. Clearly, these experiences also exposed the limitations of the current “neoliberal higher education enterprise”.

## Conclusions

This article aims to illuminate the significant shift in the higher education sector from traditional academia to neoliberal academia, with a particular focus on early and mid-career academics in our discipline, who began their careers in the 2000s and who may not fully appreciate the substantial political transformations that have occurred under neoliberalism over the past 2 decades. During this period, university cultures have undergone profound changes, primarily due to the widespread adoption of managerialism, often referred to as the "new managerialism philosophy", particularly in regions such as Australia, the UK and the USA. This shift has led to an increase in student enrolments and tuition fees, fostering a market-driven environment within higher education institutions. The proliferation of terms like "funding environment", "performance management" and "workload model" has become commonplace, contributing to an erosion of traditional academic values, including genuine scholarship, professional autonomy and discretion.

The current academic environment, often described as the "new normal", is characterised by factors such as workload allocation systems, performance management and heightened monitoring as well as bureaucracy [58]. These changes have adversely impacted the wellbeing of academics, particularly younger scholars, who may find themselves unwittingly co-opted into the managerialist agenda [2, 3, 59–61]. The demands of this new environment often divert significant time and energy away from core academic activities, such as research and teaching, and frequently conflict with the fundamental principles of autonomy and academic freedom. This can lead to distractions and confusion, particularly among early-career academics, diminishing their academic focus, productivity and wellbeing [5, 62]. The resultant cryptic pressures can contribute to a decline in wellbeing and increased stress among academic staff [42, 57, 58], exacerbated by increased competition, and low success rates for grants and fellowships from government funding bodies.

Historically, academics were regarded as intellectuals with the freedom to pursue their research, teaching and engagement interests independently. They were not recruited to serve as business managers or entrepreneurs. Contemporary evidence suggests that the current higher education system, driven by managerialism, curtails academic freedom and performance, leading to increased administrative responsibilities, heightened stress and job dissatisfaction [17, 58]. Surveys have underscored these challenges, with many academics reporting poor work-life balance, strained personal relationships and a growing desire to exit the higher education sector, particularly since the COVID-19 pandemic [57, 58]. While reversing current challenges imposed by the managerial processes and procedures now in place is difficult, it is paramount for academics to maintain and protect traditional academic values. Leaders and managers in universities need to recognise, appreciate and understand the unique academic cultures and perspectives within faculties, ensuring that academics, both young and seasoned, are respected and supported. Furthermore, from a broader perspective, governments must pay greater attention to the health, funding and support of the higher education sector and to its current financial vulnerability, as the prevailing neoliberal and managerialist approaches are proving unsustainable—based on published evidence.

Despite the pressures and challenges of the neoliberal academic environment, parasitologists continue to demonstrate remarkable resilience, creativity and dedication. Our discipline, though small and often not viewed as a national priority for research compared with areas of human medicine, makes major contributions to the fundamental understanding of parasites and parasitic diseases as well as to global animal and human health. The challenges imposed by managerialism should not weaken our resolve; instead, they should inspire us to be resourceful, navigate these complexities and seize opportunities for collaboration and innovation in the true spirit of scholarship.

Every effort must be made to create pathways for early- and mid-career researchers in Parasitology to thrive. This includes advocating for targeted funding, fostering interdisciplinary partnerships and promoting initiatives that attract investment in research, education and sustainable careers. Parasitology societies and associations have a critical role to play in driving these efforts by supporting new ventures that bring together early-career researchers and seasoned academics. Through mentorship, the fostering of collaborations and championing the discipline’s interests, these professional bodies can advocate for, and support, the development of future generations of parasitologists. By working together within collaborative frameworks—both nationally and internationally, supported by these societies and associations—we can ensure that our discipline remains strong, vibrant and impactful. Our collective strength as a community of educators and scholars will enable us to not only survive but thrive in this evolving academic landscape. By championing the work of emerging parasitologists and ensuring that they receive the support they need, we are safeguarding the future of Parasitology and its essential contributions to global health challenges.

## Data Availability

No datasets were generated or analysed during the current study.
